# Medical expenses of urban Chinese patients with stomach cancer during 2002–2011: a hospital-based multicenter retrospective study

**DOI:** 10.1186/s12885-018-4357-y

**Published:** 2018-04-17

**Authors:** Xiao-Jie Sun, Ju-Fang Shi, Lan-Wei Guo, Hui-Yao Huang, Neng-Liang Yao, Ji-Yong Gong, Ya-Wen Sun, Guo-Xiang Liu, A-Yan Mao, Xian-Zhen Liao, Ya-Na Bai, Jian-Song Ren, Xin-Yu Zhu, Jin-Yi Zhou, Ling Mai, Bing-Bing Song, Yu-Qin Liu, Lin Zhu, Ling-Bin Du, Qi Zhou, Xiao-Jing Xing, Pei-An Lou, Xiao-Hua Sun, Xiao Qi, Yuanzheng Wang, Rong Cao, Ying Ren, Li Lan, Kai Zhang, Jie He, Jia-Lin Wang, Min Dai, The listed authors are on behalf of the Health Economic Evaluation Working Group, Cancer Screening Program in Urban China (CanSPUC)

**Affiliations:** 1 School of Health Care Management (key Lab of Health Economics and Policy, National Health Commission), Shandong University, Jinan, China; 2 Office of Cancer Screening, National Cancer Center/Cancer Hospital, Chinese Academy of Medical Sciences and Peking Union Medical College, Beijing, 100021 China; 3 Department of Cancer Epidemiology, Henan Office for Cancer Control and Research, the Affiliated Cancer Hospital of Zhengzhou University, Henan Cancer Hospital, Zhengzhou, China; 4 Department of Public Health Sciences, University of Virginia, Charlottesville, VA 22908 USA; 5 Shandong Academy of Medical Sciences, Shandong Provincial Cancer Hospital Affiliated to Shandong University, Jinan, 250117 China; 6 Department of Health Economics, School of Health Management, Harbin Medical University, Harbin, China; 7 Public Health Information Research Office, Institute of Medical Information, Chinese Academy of Medical Sciences, Beijing, China; 8 Hunan Office for Cancer Control and Research, Hunan Provincial Cancer Hospital, Changsha, China; 9 Institute of Epidemiology and Health Statistics, Lanzhou University, Lanzhou, China; 10 Institute of Chronic Non-communicable Diseases Prevention and Control, Jiangsu Provincial Center for Disease Control and Prevention, Nanjing, China; 11 Department of Institute of Tumor Research, The Affiliated Cancer Hospital of Zhengzhou University, Henan Cancer Hospital, Zhengzhou, China; 12 Heilongjiang Office for Cancer Control and Research, Affiliated Cancer Hospital of Harbin Medical University, Harbin, China; 13 Cancer Epidemiology Research Center, Gansu Provincial Cancer Hospital, Lanzhou, China; 14 Teaching and Research Department, Affiliated Cancer Hospital of Xinjiang Medical University, Urumqi, China; 15 Zhejiang Office for Cancer Control and Research, Zhejiang Cancer Hospital, Hangzhou, China; 16 Chongqing Office for Cancer Control and Research, Chongqing Cancer Hospital, Chongqing, China; 17 Liaoning Office for Cancer Control and Research, Liaoning Cancer Hospital & Institute, Shenyang, China; 18 Department of Control and Prevention of Chronic Non-communicable Diseases, Xuzhou Center for Disease Control and Prevention, Xuzhou, China; 19 Ningbo Clinical Cancer Prevention Guidance Center, Ningbo NO.2 Hospital, Ningbo, China; 20 Department of Occupational Medicine, Tangshan People’s Hospital, Tangshan, China; 21 Department of Economic Operation, Kailuan General Hospital, Tangshan, China; 22 Department of Health Policy and Economic Research, Guangdong Provincial Institute of Public Health, Guangzhou, China; 23 Urban Office of Cancer Early Detection and Treatment, Tieling Central Hospital, Tieling, China; 24 Institute of Chronic disease prevention and control, Harbin Center for Disease Control and Prevention, Harbin, China; 25 Department of Cancer Prevention, National Cancer Center/Cancer Hospital, Chinese Academy of Medical Sciences and Peking Union Medical College, Beijing, China; 26 Department of Thoracic Surgery, National Cancer Center/Cancer Hospital, Chinese Academy of Medical Sciences and Peking Union Medical College, Beijing, China

**Keywords:** Stomach cancer, Medical expenses, Hospitalization, Urban, China

## Abstract

**Background:**

In China, stomach cancer is the third most common cancer and the third leading cause of cancer death. Few studies have examined Chinese stomach cancer patients’ medical expenses and their associated trends. The Cancer Screening Program in Urban China (CanSPUC) is a Major Public Health Project funded by the central government. Through this project, we have extracted patients’ medical expenses from hospital billing data to examine the costs of the first course treatments (which refers to 2 months before and 10 months after the date of cancer diagnosis) in Chinese patients with stomach cancer and the associated trends.

**Methods:**

The expense data of 14,692 urban Chinese patients with stomach cancer were collected from 40 hospitals in 13 provinces. We estimated the inflation-adjusted medical expenses per patient during 2002–2011. We described the time trends of medical expenses at the country-level, and those trends by subgroup, and analyzed the compositions of medical expenses. We constructed the Generalized Linear Mixed (GLM) regression model with Poisson distribution to examine the factors that were associated with medical expenses per patient.

**Results:**

The average medical expenses of the first course treatments were about 43,249 CNY (6851 USD) in 2011, more than twice of that in 2002. The expenses increased by an average annual rate of 7.4%. Longer stay during hospitalization and an increased number of episodes of care are the two main contributors to the expense increase. The upward trend of medical expenses was observed in almost all patient subgroups. Drug expenses accounted for over half of the medical expenses.

**Conclusions:**

The average medical expenses of the first course (2 months before and 10 months after the date of cancer diagnosis) treatments per stomach cancer patient in urban China in 2011 were doubled during the previous 10 years, and about twice as high as the per capita disposable income of urban households in the same year. Such high expenses indicate that it makes economic sense to invest in cancer prevention and control in China.

**Electronic supplementary material:**

The online version of this article (10.1186/s12885-018-4357-y) contains supplementary material, which is available to authorized users.

## Background

The population burden of cancer is high in China, and cancer has become the leading cause of death [1]. China’s share of global cancer deaths tops 25%, which is more than any other country. Stomach cancer is common in east Asian countries, and China, Japan and South Korea have reported the highest incidence rates of stomach cancer in the world [2–5]. Stomach cancer is the third most common cancer and the third leading cause of cancer death in China [6]. The age standardized incidence of stomach cancer in China in 2012 was 22.7 per 100, 000, about 5.82 times of that in the United States, and 3.15 times of that in the European Union [2]. High prevalence of *Helicobacter pylori* (HP), cigarette smoking and high intake of salt/salty food were regarded as important risk factors of stomach cancer in China [7]. A recent systematic review on global prevalence of HP infection showed that, regions with the lowest reported HP prevalence were Oceania (24.4%; 95% CI, 18.5–30.4%), Western Europe (34.3%; 95% CI, 31.3–37.2%), and Northern America (37.1%; 95% CI, 32.3–41.9%); in China, the HP prevalence was as high as 55.8% (95% CI: 51.8–59.9%) [8].

Few studies have analyzed the economic burdens of stomach cancer, especially with attention to the trends in recent years. Haga et al. showed that the medical expenses due to stomach cancer treatment accounted for approximately 10% of the medical expenses of all cancers in Japan in 2009. Additionally, they showed that the cost of treating stomach cancer has decreased from 1294 billion yen in 1996 to 1114 billion yen in 2008, largely due to decreasing incidence of stomach cancer and shortened inpatient stays [9]. Although stomach cancer is less prevalent in the U.S., in a retrospective cohort analysis of direct costs and healthcare services used in stomach cancer patients in a managed care setting, Knopf et al. found that this disease appeared to be associated with significant healthcare costs, with the largest difference shown for inpatient costs [10].

A recent systematic review showed that the data about economic burden of cancer in China were still very limited, with relatively poor comparability [11]. Some small-scale studies in China analyzed the economic burden of stomach cancer. Based on the data of 1957 stomach cancer patients discharged in 2014 in Shanxi province, Li et al. found that, controlling for other factors, receiving surgery, length of stay, hospital’s specialty type, region of residence, and age were significantly associated with inpatient expenses [12]. Cun et al. analyzed the inpatient medical expenses of 3287 stomach cancer patients in a general hospital of Shanxi province from 2002 to 2008, and found that inpatient expenses increased at an annual rate of 13.5%, and insured patients had much higher inpatient expenses than uninsured patients [13]. Another recent systematic review concluded that the evidence for economic burden of stomach cancer in China was still limited over the past two decades and mainly focused on individual and regional levels. And an increase and differences in provinces were observed in direct medical expenditures [14].

In China, health-care facilities mainly include hospitals, grassroots medical institutions and public health institutions. Tertiary hospitals are the highest level of hospital and include national, provincial, municipal and medical-school-affiliated hospitals. By 2012, there were 1624 tertiary hospitals and 6566 secondary hospitals in China, which were classified as general hospitals, traditional Chinese medicine (TCM) hospitals, Chinese and allopathic medicine hospitals, ethnic medicine hospitals, specialized hospitals or nursing homes [15]. By 2011, China had more than 200 cancer hospitals, with more than 30 tertiary-level hospitals for cancer that provided the highest level of care [16]. In addition, many general hospitals have oncology departments. Because patients with cancer are free to choose their health-care provider, they tend to gravitate to large tertiary hospitals in urban areas [17]. This trend prolongs time to diagnosis and treatment, and increases the likelihood of out-of-pocket expenses for patients [18].

In name, China’s single payer system has achieved universal health coverage in recent years, but high co-pay and co-insurance cause cancer patients to pay high out-of-pocket expenses [19]. The risk of catastrophic out-of-pocket expenditure drove 12.9% of households into poverty in 2011 [20], and patients with cancer were particularly at risk [21]. In 2009, to ensure access to essential medicines for poor and uninsured patients, China released the National Essential Drugs List. This list is a catalogue of drugs that are priced at the manufacturer’s cost without additional fees for profit and has higher reimbursement rates available under the national insurance system than for other drugs [22]. As of 2012, only 24 anticancer drugs (and only one opioid analgesic) were included in the list [23]. Innovative drugs are often unavailable through the National Essential Drugs List or local Reimbursement Drug Lists, so patients have to cover the full cost of these expensive drugs [23, 24]. Additionally, some patients might be overtreated through off-label use of anticancer drugs [25]. Since 2012, China’s Ministry of Health began to lower the co-pay and co-insurance for certain chronic diseases of high economic burden, including stomach cancer and seven kinds of other cancer [26]. However, the co-insurance of stomach cancer treatments is still around 30% [27].

Few studies have examined the medical expenses of Chinese patients with stomach cancer and their associated trends. We use national-level data to address this knowledge gap and important health policy question. The Cancer Screening Program in Urban China (CanSPUC), started in August of 2012, is a Major Public Health Project funded by the central government. Through this project, we have extracted patients’ medical expenses from hospital billing data to examine the costs of the first course treatments in Chinese patients with stomach cancer and the associated trends.

## Methods

### Data source

Sampled hospitals collected and reported patient-level data of their demographics, clinical information, and medical expenses of the first course treatments which refers to 2 months before and 10 months after the date of cancer diagnosis [28, 29]. In this study, only the medical expenses in the main treatment hospital, where most of treatment expenses for one patient were spent, were collected. The medical expenses for cancer treatments in the reporting hospital included payments (both of out-of-pocket payments and payments by insurance plans) of each patient for admissions and outpatients from the first admission date to the last discharge date (one patient may have more than one hospital admissions in this hospital). Hospital admissions for non-cancer conditions (not diagnosed with stomach cancer or with suspicion of stomach cancer) are excluded in the expense analysis. Expense items were grouped into five categories: drug costs, surgery and treatment, diagnostic tests, nursing care, and other. The national consumer price index (CPI) of healthcare and personal articles (e.g. medicine, healthcare appliances and cosmetics) was used to adjust for inflation in medical expenses during the study period, and we calculated our estimates in terms of 2011 Chinese yuan (CNY). Expense trends were calculated based on estimates for successive calendar years. For patients whose care spanned more than one calendar year, all expenses were assigned to the year of the last discharge.

### Sample selection

Patients were treated at 40 general or cancer hospitals in 22 cities including Beijing, Dongguan, Foshan, Guangzhou, Shenzhen, Zhongshan, Jinan, Nantong, Xuzhou, Shenyang, Tieling, Hangzhou, Ningbo, Tangshan, Zhengzhou, Harbin, Daqing, Changsha, Urumchi, Lanzhou, Jinchang, and Chongqing. These cities vary by their geographic region and level of economic development.

Patients’ data were collected if: (1) a patient was diagnosed with first primary stomach cancer; (2) his or her residence was registered as urban; (3) the time of the last discharge was between January 1, 2002 and December 31, 2011; (4) the patient had been hospitalized for major cancer treatments in the reporting hospital (most Chinese cancer patients receive cancer treatments at inpatient settings, as the unique reimbursement policy of China’s public insurance usually does not pay for outpatient services); (5) the patient’s demographic information and expense data were available, (6) and their tumor factors and treatments data were relatively complete.

In order to collect data from a balanced sample, we used stratified sampling by selecting samples independently within strata by province of residence, year of diagnosis, sex, and stage at diagnosis. Starting with an initial sample size of 14,692, we later excluded cases without definite dates of diagnosis and/or discharge. Those cases with extreme values were also excluded, resulting in a final analysis sample size of 14,297.

### Statistical analysis

We firstly described the basic characteristics of study cities, hospitals, and stomach cancer cases. Next, we estimated the average total treatment expenses after CPI adjustments in terms of the country-level sample and the provincial-level samples. The natural logarithm conversion was performed for those expense indicators with high discrete degrees. A two-sample test for independent samples was used between two groups; a one-way ANOVA test was used to compare the differences among three and above groups, and the Student-Newman-Keuls Test (SNK Test) was used to compare the differences within groups. In this study, *p*-values of less than 0.05 (two-sided test) were considered statistically significant. We also described the time trends of medical expenses during 2002–2011 at the country-level, and those trends by subgroup (area, gender, stage, and so on), and computed the Average Tempo to estimate the average annual growth rates of medical expenses.

To examine the factors that were associated with medical expenses per patient, we constructed the Generalized Linear model (GLM) with Poisson and Gamma distributions, as suggested by Manning and colleagues for expenditure data [30]. Independent variables are age at diagnosis, gender, region of residence, hospital’s specialty type, hospital accreditation level, tumor stage, cancer treatments, number of episodes of care (a single outpatient visit or hospital stay), the year of last discharge, number of inpatient days, and if the patient has comorbidities. Finally, we compared and analyzed the compositions of medical expenses from 2002 to 2011.

Subgroup analyses were performed to assess the robustness of the time trend of medical expenses in different provinces, province groups, and patient groups by tumor stage and treatments received. The data was analyzed using SAS 9.3 software.

### Ethics statement

The study protocol of the CanSPUC was reviewed and approved by the Institutional Review Board of the Cancer Institute & Hospital, Chinese Academy of Medical Sciences.

## Results

Table 1 describes the basic information of the provinces, cities, and hospitals that were involved in this study. These provinces differ by population size and per capita disposable income of urban residents. The population sizes of Guangdong, Shandong, and Henan were about 100 million each, while the numbers in Xinjiang, Gansu, and Chongqing were between 20 to 30 million. The per capita disposable income in Beijing, Zhejiang, Jiangsu, and Guangdong were above 25,000 CNY (3960 USD), but it was only 14,989 CNY (2374 USD) in Gansu. Over half of sampled hospitals are general hospitals (19, 56%), and only about one fifth of hospitals were non-tertiary (below level 3A) hospitals.

**Table 1:** Survey sites and hospitals in 13 provinces

Province	General information		Study sites and hospitals involved		
	Population size in 2011^a^, 1000	Per capita disposable income of urban residents in 2011^a^, CNY	City or cities involved	Number of general hospital involved (level 3A)^b^	Number of cancer hospitals involved (level 3A)^b^
Beijing	20,190	32,903	Beijing	2 (Yes) 1 (No)	2 (Yes)
Zhejiang	54,630	30,971	Hangzhou, Ningbo	1 (Yes)	1 (Yes)
Guangdong	105,050	26,897	Guangzhou, Shenzhen, Dongguan, Foshan, Zhongshan	5 (Yes)	0
Jiangsu	78,990	26,341	Nantong, Xuzhou	1 (No)	2 (Yes)
Shandong	96,370	22,792	Jinan	0	1 (Yes)
Liaoning	43,830	20,467	Shenyang, Tieling	1 (Yes)	1 (Yes)
Chongqing	29,190	20,250	Chongqing	0	1 (Yes)
Hunan	65,960	18,844	Changsha	0	1 (Yes)
Hebei	72,410	18,292	Tangshan	2 (Yes)	0
Henan	93,880	18,195	Zhengzhou	0	1 (Yes)
Heilongjiang	38,340	15,696	Harbin, Daqing	2 (Yes)	2 (Yes)
Xinjiang	22,090	15,514	Urumchi	0	1 (Yes)
Gansu	25,640	14,989	Lanzhou, Jinchang	4 (No)	1 (Yes) 1 (No)
Total	–	–	22	19	15

Note: ^a^ Based on China Statistical Yearbook 2012, available from http://www.stats.gov.cn/tjsj/ndsj/2012/indexch.htm

^b^ Hospitals in China are organized according to a 3-tier system that recognizes a hospital’s ability to provide medical care, medical education, and conduct medical research. Based on this, hospitals are designated as Primary, Secondary or Tertiary institutions. Further, based on the level of service provision, size, medical technology, medical equipment, and management and medical quality, these 3 grades are further subdivided into 3 subsidiary levels: A, B and C. This results in a total of 9 levels. 3A is the highest grade/level in the hospital classification

Table 2 presents descriptive statistics for the sample. About half of the cancer patients were from eastern China, more than two thirds of patients were treated in cancer hospitals, and the majority of them (93%) were treated in 3A-level hospitals. The proportion of male patients was 41% higher than that of female patients. The average age of sampled patients was 58 years old, with 33% of them aged 65 or older. The proportion of stage IV patients (34%) was about two times that of stage I patients, and 7% of patients were upstaged. The majority of patients (79%) had adenocarcinoma. Most patients (68%) had only one hospitalization. The median of inpatient days was 23 days. 41% of patients have received surgery only, and 25% received both surgery and chemotherapy. About one third of patients had co-morbidities, and 11% of patients had complications after cancer treatments.

**Table 2:** Characteristics of stomach cancer cases (*n* = 14,297), 2002–2011

Variable		
Region of residence, n (%)		
East	7297	(51.0)
Central	3744	(26.2)
West	3256	(22.8)
Hospital type, n (%)		
General	4220	(29.5)
Specialized	10,077	(70.5)
3A level hospital, n (%)		
Yes	13,223	(92.5)
No	1074	(7.5)
Gender		
Male	10,092	(70.6)
Female	4205	(29.4)
Age at diagnosis, y, mean ± SD	58.1 ± 12.6	
Age at diagnosis, y		
< 45	2119	(14.8)
45~ 54	3167	(22.2)
55~ 64	4337	(30.3)
≥ 65	4674	(32.7)
Pathological type		
Adenocarcinoma	11,270	(78.8)
Others	2525	(17.7)
Unknown	502	(3.5)
Clinical stage		
I	2357	(16.5)
II	2590	(18.1)
III	3452	(24.1)
IV	4838	(33.8)
Unknown	1060	(7.4)
The proportion of morphological verification, %	12,632	(88.4)
Number of episodes per patient, Median (P5-P95)	1 (1–6)	
Number of episodes per patient		
1	9667	(67.6)
2	1876	(13.1)
3	1011	(7.1)
4+	1743	(12.2)
Number of inpatient days per patient, Median (P25-P75)	23 (15–38)	
Type of therapy		
Surgery	5793	(40.5)
Surgery & Chemotherapy	3555	(24.9)
Surgery & Radiotherapy	50	(0.3)
Chemotherapy	2386	(16.7)
Radiotherapy	314	(2.2)
Radiotherapy & Chemotherapy	328	(2.3)
Palliative care	1438	(10.1)
Others	372	(2.6)
Unknown	61	(0.4)
% of patients with any co-morbidities	4666	(32.6)
% of patients with any complications	1528	(10.7)

### Medical expenses in patient subgroups

Table 3 shows that the medical expenses of the first course treatments in stomach cancer patients differ by region of residence, hospital’s specialty type, hospital accreditation level, number of episodes of care, and tumor pathology and stage. The medical expenses per patient in the eastern and central provinces were nearly 50% higher than that in the western provinces. Medical expenses per patient in cancer hospitals were 11% higher than that of general hospitals, and 3A-level hospitals were 59% higher than lower-level hospitals. The expenses increased as the number of episodes of care increases. Expenses of adenocarcinoma patients were 34% higher than that of the others. Although the medical expenses differed significantly by tumor stage, the differences are not substantial.

**Table 3:** Medical expense for stomach cancer diagnosis and treatment per patient

Variable	Expense per patient during 2002–2011, CNY Mean (95% CI)	*P-value*
Overall	32,403 (31,953–32,854)	
Region		< 0.001^a^
East	37,901(37,149–38,653)	
Central	34,850 (34,164–35,536)	
West	23,794 (22,876–24,712)	
Hospital type		< 0.001^b^
General hospital	31,496(30,666–32,326)	
Specialized hospital	33,342 (32,791–33,892)	
3A hospital		< 0.001^b^
Yes	33,330 (32,862–33,799)	
No	20,989 (19,492–22,485)	
Number of hospitalizations per patient		< 0.001^a^
1	24,928 (24,526–25,330)	
2	37,620 (36,400–38,839)	
3	47,462 (45,377–49,547)	
4+	59,513 (57,823–61,203)	
Gender		0.956^b^
Male	32,506 (31,966–33,046)	
Female	32,156 (31,340–32,973)	
Age at diagnosis, y		0.639^a^
< 45	32,046 (30,869–33,223)	
45~ 54	32,371 (31,406–33,336)	
55~ 64	31,542 (30,771–32,314)	
≥ 65	33,386 (32,564–34,208)	
Pathological type		< 0.001^b^
Adenocarcinoma	34,553 (34,033–35,074)	
Others	25,704 (24,813–26,596)	
Clinical stage		< 0.001^a^
I	30,306 (29,392–31,219)	
II	30,158 (29,251–31,064)	
III	34,039 (33,121–34,958)	
IV	32,939 (32,106–33,773)	

Note: ^a^ ANOVA test after logarithm transition

^b^ Two-sample Student t test after logarithm transition

**Fig. 1:**
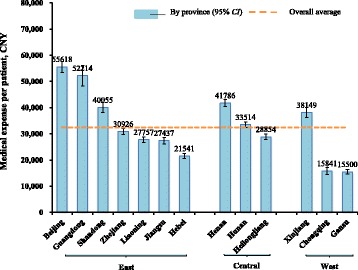
Medical expense for stomach cancer diagnosis and treatment per patient, by province

Figure 1 shows regional differences in medical expenses of the first course treatments. Beijing, Guangdong, Shandong, Xinjiang, Hunan and Henan spent over the national average (32,403 CNY, 95% CI: 31,953–32,854). The medical expenses per patient in Beijing were 3.5 and 3.6 times of those in Chongqing and Gansu, respectively.

Results from the Generalized Linear Mixed (GLM) regression model with Poisson distribution echo the findings from the subgroup analyses. We also found age at diagnosis, drug proportion, number of episodes of care, number of inpatient days, comorbidities, year of last discharge, region of residence, hospital’s specialty type, hospital accreditation level, and tumor pathology and stage were statistically significant in their association with medical expenses (Table 4).

**Table 4:** Generalized linear model (GLM)-Poisson regression model

Parameter	Univariate analysis		Multivariate analysis	
	Relative risk (RR)	95% CI	RR	95% CI
Intercept	–	–	3036.012^**^	3033.281–3038.745
Age at diagnosis (45~ 54 VS < 45)	1.010^**^	1.010–1.010	1.029^**^	1.028–1.029
Age at diagnosis (55~ 64 VS < 45)	0.984^**^	0.984–0.985	1.006^**^	1.006–1.006
Age at diagnosis (65~ VS < 45)	1.042^**^	1.042–1.042	1.113^**^	1.112–1.113
Region (East VS West)	1.470^**^	1.470–1.471	1.071^**^	1.071–1.072
Region (Central VS West)	1.465^**^	1.464–1.465	1.116^**^	1.115–1.116
Hospital level (3A VS Non-3A)	1.588^**^	1.587–1.589	1.383^**^	1.382–1.383
Hospital type (Specialized VS General)	1.105^**^	1.105–1.106	1.013^**^	1.013–1.013
Drug proportion (%)	1.009^**^	1.009–1.009	1.009^**^	1.009–1.009
Clinical stage (II VS I)^a^	0.995^**^	0.995–0.995	0.952 ^**^	0.952–0.952
Clinical stage (III VS I)^b^	1.123^**^	1.123–1.124	1.018^**^	1.018–1.018
Clinical stage (IV VS I)	1.087^**^	1.087–1.087	1.079^**^	1.079–1.079
Type of therapy (Surgery VS Palliative care)	2.323^**^	2.322–2.324	2.330^**^	2.328–2.331
Type of therapy (Surgery & Chemotherapy VS Palliative care)	3.223^**^	3.222–3.225	2.129^**^	2.128–2.130
Type of therapy (Surgery & Radiotherapy VS Palliative care)	2.990^**^	2.986–2.995	2.014^**^	2.011–2.017
Type of therapy (Chemotherapy VS Palliative care)	1.909^**^	1.908–1.910	1.436^**^	1.435–1.436
Type of therapy (Radiotherapy VS Palliative care)^c^	1.103^**^	1.102–1.104	1.166^**^	1.165–1.167
Type of therapy (Radiotherapy & Chemotherapy VS Palliative care)	2.758^**^	2.756–2.760	1.962^**^	1.961–1.963
Type of therapy (Others VS Palliative care)	0.856^**^	0.855–0.857	0.880^**^	0.880–0.882
Number of episodes per patient	1.201^**^	1.201–1.201	1.101^**^	1.101–1.101
Year (2003 VS 2002)	1.146^**^	1.146–1.147	1.107^**^	1.106–1.108
Year (2004 VS 2002)	1.328^**^	1.327–1.328	1.198^**^	1.197–1.199
Year (2005 VS 2002)	1.481^**^	1.480–1.482	1.316^**^	1.315–1.316
Year (2006 VS 2002)	1.391^**^	1.391–1.392	1.230^**^	1.229–1.230
Year (2007 VS 2002)	1.535^**^	1.534–1.535	1.273^**^	1.273–1.274
Year (2008 VS 2002)	1.614^**^	1.614–1.615	1.266^**^	1.266–1.267
Year (2009 VS 2002)	1.674^**^	1.673–1.675	1.415^**^	1.414–1.415
Year (2010 VS 2002)	1.792^**^	1.791–1.793	1.499^**^	1.499–1.500
Year (2011 VS 2002)	2.049^**^	2.048–2.050	1.579 ^**^	1.578–1.580
Number of inpatient days per patient	1.008^**^	1.008–1.008	1.006 ^**^	1.006–1.006
Accompanying diseases (Yes VS No)	1.220^**^	1.220–1.221	1.188^**^	1.188–1.188

Note: ^a^ **P < 0.01

^b^ We include link = log. When we write our model out, log (μ) = β_0_ + β_1_x_1_ + ... + β_p_x_p_, where μ is the count we are modeling, and log ( ) defines the link function (i.e., how we transform μ to write it as a linear combination of the predictor variables)

^c^ Goodness of fit for the multivariate GLM model: deviance = 0.3437 (*p* ≈ 1), Pearson Χ^2^ = 1.255(*p* ≈ 1), so the goodness of fit for the model is very good

### Time trends of medical expenses and other important measures (2002–2011)

**Fig. 2:**
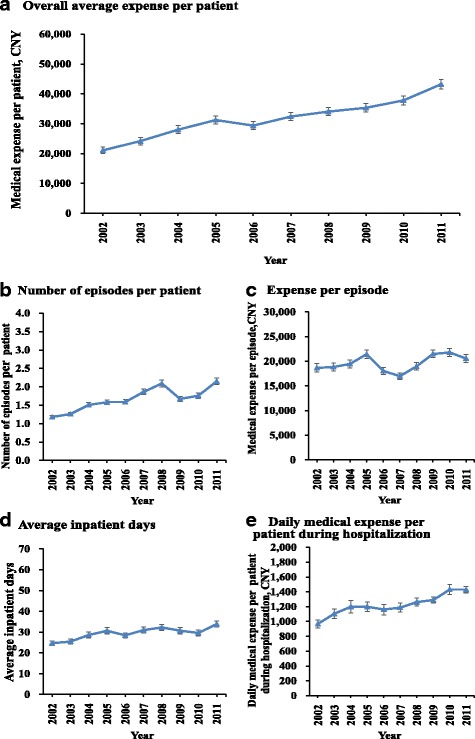
Time trends of medical expenses and other important measures (2002–2011). **a** to **e** show the time trends of medical expense per patient (**a**), number of episodes (**b**), expense per episode (**c**), average inpatient days (**d**), daily medical expense per patient during hospitalization (**e**)

Figure 2 shows that medical expenses of the first course treatments per patient increased steadily by an average annual rate of 7.4% (Fig. 2a). In 2011, medical expenses were 2 times as much as those in 2002. Except for 2006, the rising trend was seen in all preceding years. The number of episodes of care per patient increased over time (Fig. 2b). The expenses per episode of care did not present a definite changing trend, although it declined by 21% from 2005 to 2007 and rose back by 26% in 2009 (Fig. 2c). The time trend of inpatient days per patient grew slowly in fluctuations (Fig. 2d). In 2011, it was 1.4 times as long as that in 2002. Also, except for 2006, 2009 and 2010, the rising trend was observed in all the other years. During the study period, the daily average hospitalization expenses per patient presented a slowly increasing trend (4% annually) (Fig. 2e).

### Time trend of medical expenses by patient subgroups (2002–2011)

**Fig. 3:**
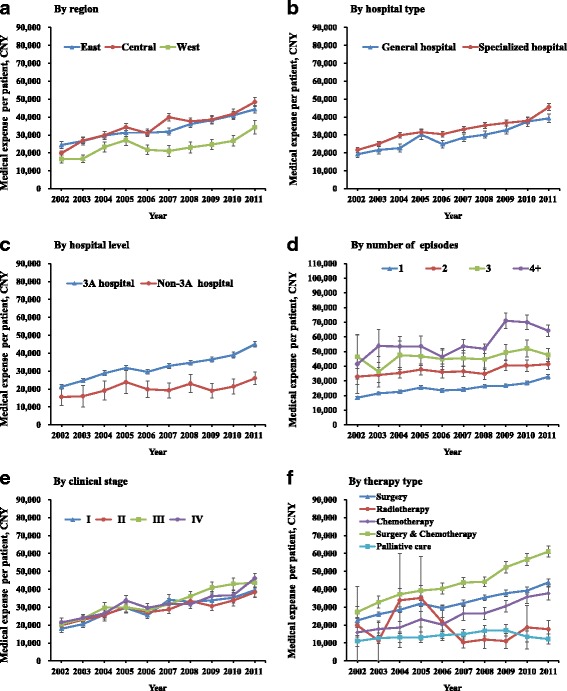
Yearly trend of medical expense for stomach cancer diagnosis and treatment per patient, by subgroup, 2002–2011. **a** to **f** show the yearly trend of medical expense per patient by region (**a**), by hospital type, (**b**) by hospital level (**c**), by number of episodes (**d**), by clinical stage (**e**), by therapy type (**f**)

Figure 3 shows that the upward trend of medical expenses of the first course treatments was observed in almost all patient subgroups, and fluctuations were only seen in patient strata with small sample sizes. For example, in 2002, the medical expenses per patient in the central provinces were 1.2 times of that in the western provinces; 5 years later, the gap expanded to 1.9 times; and in 2011, the gap was reduced to 1.4 times (Fig. 3a). From Fig. 3f, we can see that, except small drops in 2006, the medical expenses per patient among those receiving “chemotherapy” and “surgery and chemotherapy” presented a steadily upward trend, increasing by the average annual growth rates of 6.9 and 8.4%, respectively. However, for those receiving “radiotherapy,” it dramatically increased by 198.6% from 2003 to 2004, and steeply declined by 70.6% from 2005 to 2007.

### The compositional changes of medical expenses (2002–2011)

**Fig. 4:**
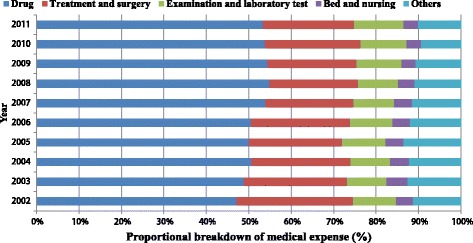
The breakdown of medical expense for stomach cancer diagnosis and treatment per patient, 2002–2011. Others include fees for diagnosis, registration, oxygen, blood and heating

During the 10 years, the proportion of drug expenses was always the most substantial, and increased from 46% in 2002 to 53% in 2011. The proportion of “treatment and surgery fees” declined by 6% from 2002 to 2011. The proportion of inspection and laboratory test fees increased by 1%, and there was a 0.5% decrease in bed and nursing fees (Fig. 4).

### Subgroup analyses

(Additional file 1: Figure S1) summarizes the findings of subgroup analysis on medical expenses of the first course treatments by province groups. It was found to be quite sensitive to the province selected (Additional file 1: Figure S1A). Further analysis indicated that some provinces (Beijing, Shandong, Guangdong, Jiangsu, Henan, and Xinjiang) had better data quality in terms of the completeness of number of episodes of care. Additional file 1: Figure S1B shows that medical expenses per patient were somewhat sensitive to province groups by data completeness, and the overall medical expenses per patient may be underestimated to some extent because of incomplete data from some provinces. However, the upward trends of medical expenses per patient in two scenarios were still similar to each other.

(Additional file 2: Table S1) shows medical expenses by tumor stage and treatments that patients received.

(Additional file 3: Table S2) shows the results from the Generalized Linear Mixed (GLM) regression model with Gamma distribution.

## Discussion

Cancer is one of the most expensive illnesses. This is true in both developed and developing countries. This study showed that the average medical expenses of the first course treatments per stomach cancer patient in urban China were as high as 43,249 CNY (6851 USD) in 2011. A recent national study in China showed that the incidence rate of stomach cancer for males only decreased by 1.8% from 2003 to 2011, and for females, it only decreased by 2.7% from 2000 to 2011. It was predicted that there would be about 235,200 and 444, 000 newly diagnosed stomach cancer cases in 2015 in urban and rural China, respectively [31]. This study showed that the expenses of the first course treatments per patient increased at a yearly rate of 7.4%, close to the average growth rate (10.2%) of real per capita Gross Domestic Product (GDP) at the same time period [32]. With the improvement of early diagnosis of stomach cancer in China, the incidence rates in the near future may not decline obviously; at the same time, with more innovative or imported expensive chemotherapeutics introduced, treatment effects would be further improved, and drug expenses would also be escalated. The average medical expenses per stomach cancer patient were doubled compared to the number in 2002, and about twice as high as the per capita disposable income of urban households in 2011. According to data on average family population (2.9) and the per capita disposable income (21,809.8 CNY) of urban households in 2011 [32], the average medical expenses of the first course treatments per stomach cancer patient were 68.4% of the average family annual income of urban households. A study in a relatively affluent area in Pakistan showed that the ratio of cancer care expenses to family income was about 1.1 in 2010 [33]. Another study in Mexico found that the average direct medical cost/year of care in the public sector for a woman with breast cancer was $8557 in 2009 [34], which is very close to the Gross National Income (GNI) per capita ($8960) over the same period [35]. However, the total medical care cost per cancer case in the U.S. in 2011 was $11,501.7 [36], only accounting for 23.7% of the GNI per capita ($48,620) [37].

There were 198,000 and 226,000 newly diagnosed stomach cancer cases in 2012 in rural and urban China, respectively [38]. If we believe the medical expenses of the first course treatments in this paper are somewhat underestimated for urban patients, and rural Chinese patients had slightly lower expenses than their urban counterparts, it is probably safe to use 6851 USD both for urban and rural patients as the average yearly expense of the first course of treatments. And then, it can be estimated that it costs China around $3 billion USD in 2012 to treat stomach cancer during the first course treatments. With an aging population, increased pollution from overheated development, an adopted Western lifestyle characterized by sedentary behavior, high-fat diets and obesity closely related with high body fatness [39], and a high volume of tobacco and alcohol use, which have been confirmed as risk factors of gastric cancer with “convincing” or “limited” evidence [40, 41], we do not expect a rapid decrease of stomach cancer cases. Therefore, with rising expenses per gastric cancer patient, we might not see reduction of care expenses for gastric cancer.

In this study, we found that, during 2002–2011, the medical expenses of the first course treatments per patient among those receiving “chemotherapy” and “surgery and chemotherapy” presented a steadily upward trend, except in 2006. In another study in Shanxi province of China, Cun et al. also found such a fall of hospitalization expenses in 2006 [13]. The “2006 Statistical Bulletin of National Health Development” also showed that, the growth range of medical expenses in 2006 was the lowest during the past 20 years [42]. This may explain the above expense drops in 2006. We also found that there were steep increase and decrease of radiotherapy expenses from 2003 to 2007, which was mainly caused by the obviously bigger variations (2004, 95% CI: 7622–60022; 2005, 95% CI: 11809–58398; 2006, 95% CI: 12378–31151) resulted from the smaller sample sizes of cases receiving radiotherapy during 2004–2006.

We were concerned about the possibility that some patients were treated in multiple hospitals, which greatly harms the completeness of the study data. Because there are more treatment modalities available for early stage gastric cancer patients than late stage cases [43], we expect that early stage cases have higher odds of receiving cancer treatments at multiple hospitals. Therefore, we feel that the medical expenses of stage I and II patients could be underestimated relative to late stage cases. Our future study will use health insurance claims data to address this issue. A separate paper will be written in the future to discuss the medical expenses by tumor stage and treatments.

The well-known slogan “kanbing nan, kanbinggui”, which means “it is difficult and expensive to seek medical treatment”, aptly describes the situation for Chinese cancer patients. A recent abstract in the lancet showed that out-of-pocket expenditure of newly diagnosed cancer (2 months before and 10 months after diagnosis) per cancer patient in urban China was $4947 (4875–5020), accounting for 57.5% of annual household income, presenting 77.6% of families with an unmanageable financial burden [44]. With a high co-insurance rate in China, it is always a struggle for cancer patients to decide between spending savings intended for their children’s or grandchildren’s education, selling property on which the family’s livelihood depends, or borrowing money from relatives and friends for a cure that is often elusive. It is a challenge for the Chinese government to manage cancer burden effectively and efficiently. The nature of disease in China has changed from a primary burden of infectious disease to a burden dominated by chronic, non-communicable diseases such as cancer. It therefore makes economic sense to invest in global cancer prevention and control, especially in low- and middle-income countries.

Although a number of factors influence the expenses of cancer care, escalating cancer drug expenses are probably responsible for a substantial proportion of the skyrocketed cancer care expenses. Some of these new treatments provide only marginal survival benefit. Like the U.S. Food and Drug Administration (FDA), the Chinese FDA approves cancer drugs based on drug safety and efficacy, but it does not consider costs or cost-effectiveness in its decisions. As new, more expensive treatments (immunotherapy and targeted therapy) and other new technologies become the standard of care in the near future, the expenses of cancer care will rise rapidly.

Several limitations should be noted with this study. Firstly, in this study, limited by available time, funds and human resources, we only collected the medical expense information in the hospitals that cancer patients received their most treatment, which didn’t include their expense information from other medical institutions or self-treatments. So, the medical expense of the first course treatments per patient may be underestimated to some extent. Secondly, the survey on medical expense was done in the sites which undertook clinical screenings arranged by the CanSPUC project, which didn’t involve those hospitals in small cities. Therefore, we couldn’t estimate the medical expense of the first course treatments per patient for those who received their most treatment in small cities. Thirdly, in this study, we didn’t get the insurance reimbursement information, so it was impossible to analyze the actual economic burdens from out-of-pocket payments among stomach cancer patients during 2002–2011. Fourthly, the widespread differences in medical expenses between regions may be related to accessibility differences to treatment, which should be further explored in the future. In addition, and the majority of sample hospitals in this study were 3A hospitals (3A is the highest level in the hospital classification in China), which followed internationally recommended cancer treatment guidelines better than lower-level hospitals, so the differences of treatment modalities between different areas in this study were not so obvious. Fifthly, although the CPI growth rates among different provinces may have small differences (for example, in 2012, the CPI growth rates in sample provinces in this study ranged from 1.46 to 3.16%, and the national CPI growth rate was 2.03%) [32], a national CPI number was used in this study because we couldn’t get all the selected provinces’ CPIs of medical care during 2002–2011, so it is challenging to separate the trend changes from price changes on regional levels. Finally, data used in this study were during 2002–2011, which look like somewhat old; however, it still has important values, because it is the first national-level trend analysis of medical expenses of urban Chinese patients with stomach cancer, and it will set a good base for future comparisons with future findings based on newer data.

## Conclusions

The average medical expenses of the first course treatments in urban Chinese stomach cancer patients in 2011 were doubled during the previous 10 years, and about twice as high as the per capita disposable income of urban households in the same year. Such high expenses indicate that it makes economic sense to invest in cancer prevention and control in China.

## Additional files

Additional file 1: **Figure S1.** Yearly trend of medical expense for stomach cancer diagnosis and treatment per patient, by province groups, 2002-2011 . A to B show the yearly trend of  medical expense per patient  by province (A), by data reliability (B). (PDF 418 kb)

Additional file 2: **Table S1.** Medical expenses for stomach cancer diagnosis and treatment per patient by stage and therapy (average expenses, 95%CI). (DOCX 15 kb)

Additional file 3: **Table S2.** Generalized linear model (GLM)-Gamma regression model. (DOCX 18 kb)

## Abbreviations

CanSPUC: the Cancer Screening Program in Urban China
CNY: Chinese yuan
CPI: consumer price index
FDA: Food and Drug Administration
GDP: Gross Domestic Product
GLM: Generalized Linear Mixed
GNI: Gross National Income
HP: *Helicobacter pylori*
SNK: Student-Newman-Keuls
TCM: Traditional Chinese medicine
