# Supplementary figures and images for: Medical expenses of urban Chinese patients with stomach cancer during 2002–2011: a hospital-based multicenter retrospective study
Source: BMC Cancer. 2018 Apr 17;18:435. doi: 10.1186/s12885-018-4357-y (PMC5905135; doi:10.1186/s12885-018-4357-y)

### A. By province

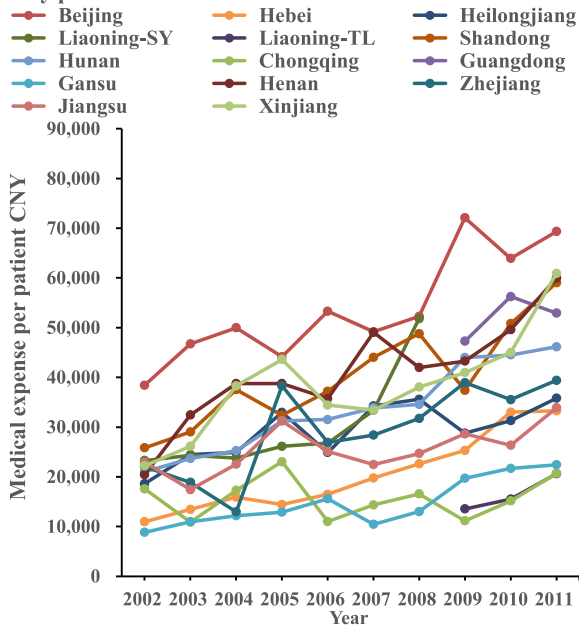

### B. By data reliability

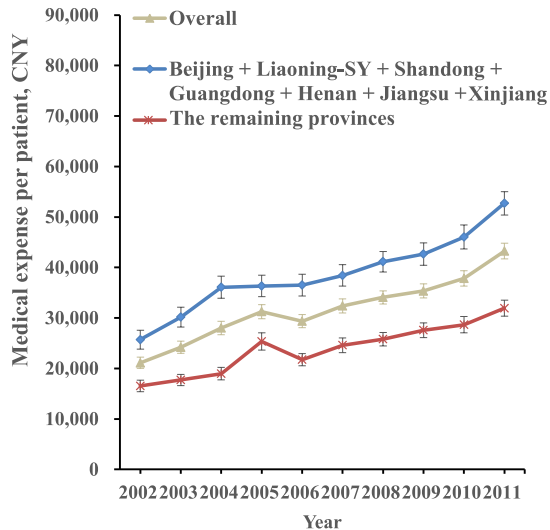

Supplement: Supplementary file 1 — Figure S1. Yearly trend of medical expense for stomach cancer diagnosis and treatment per patient, by province groups, 2002-2011 . A to B show the yearly trend of medical expense per patient by province (A), by data reliability (B). (PDF 418 kb) [file 12885_2018_4357_MOESM1_ESM.pdf]
