# Supplementary material for: Medical expenses of urban Chinese patients with stomach cancer during 2002–2011: a hospital-based multicenter retrospective study
Source: BMC Cancer. 2018 Apr 17;18:435. doi: 10.1186/s12885-018-4357-y (PMC5905135; doi:10.1186/s12885-018-4357-y)
Supplement: Supplementary file 2 — Table S1. Medical expenses for stomach cancer diagnosis and treatment per patient by stage and therapy (average expenses, 95%CI). (DOCX 15 kb) [file 12885_2018_4357_MOESM2_ESM.docx]

**Table S1 Medical expenses for stomach cancer diagnosis and treatment per patient by stage and therapy (average expenses, 95%CI)**

| Type of therapy | Stage I | Stage II | Stage III | Stage IV |
| --- | --- | --- | --- | --- |
| Surgery | 32,451 (31,420-33,482) | 29520 (28,525-30,515) | 32,124 (31,081-33,166) | 33,048 (31,869-34,227) |
| Surgery & Chemotherapy | 42,691 (40,272-45,110) | 40,940  (39,206-42,854) | 45,138  (43,356-46,921) | 47,571 (45,610-49,531) |
| Surgery & Radiotherapy | 20,743* | 42,888  (26,691-59,086) | 33,967  (9,007-58,927) | 49,815 (33,103-66,527) |
| Chemotherapy | 19,645 (16,942-22,349) | 22,069 (19,538-24600) | 23,741 (21,067-26,416) | 31,320 (29,593-33,047) |
| Radiotherapy | 11,878 (7,148-16,609) | 16,460  (12,095-20,825) | 9,215  (6,676-11,754) | 20,046 (15,203-24,890) |
| Radiotherapy & Chemotherapy | 32,570 (19,944-45,196) | 39,605 (28,489-50,721) | 31,073  (22,471-39,675) | 42,556 (36,175-48,938) |
| Palliative care | 11,351 (9,602-13,100) | 12,225 (10,406-14,044) | 13,105 (10,718-15,493) | 15,295 (14,940-16,651) |
| Others | 7, 262  (4,670-9,853) | 10,741 (7,974-13,509) | 19,609 (10,590-28,629) | 13,051 (10,531-15,571) |

*n=1
