# Supplementary material for: Medical expenses of urban Chinese patients with stomach cancer during 2002–2011: a hospital-based multicenter retrospective study
Source: BMC Cancer. 2018 Apr 17;18:435. doi: 10.1186/s12885-018-4357-y (PMC5905135; doi:10.1186/s12885-018-4357-y)
Supplement: Supplementary file 3 — Table S2. Generalized linear model (GLM)-Gamma regression model. (DOCX 18 kb) [file 12885_2018_4357_MOESM3_ESM.docx]

**Table S2 Generalized linear model (GLM)-Gamma regression model**

| **Parameter** | **Multivariate analysis** | |
| --- | --- | --- |
|  | **RR** | **95% CI** |
| Intercept | 2279.474** | 2104.641-2468.584 |
| Age at diagnosis (45~54 VS <45) | 1.037* | 1.002-1.072 |
| Age at diagnosis (55~64 VS <45) | 1.043* | 1.010-1.077 |
| Age at diagnosis (65~ VS <45) | 1.134** | 1.098-1.171 |
| Region (East VS West) | 1.248** | 1.212-1.284 |
| Region (Central VS West) | 1.270 ** | 1.229-1.313 |
| Hospital level (3A VS Non-3A) | 1.409 ** | 1.341-1.482 |
| Hospital type (Specialized VS General) | 1.045** | 1.019-1.072 |
| Drug proportion (%) | 1.009** | 1.008-1.009 |
| Clinical stage (II VS I)^a^ | 0.973 | 0.941-1.006 |
| Clinical stage (III VS I)^b^ | 1.015 | 0.983-1.047 |
| Clinical stage (IV VS I) | 1.073** | 1.041-1.106 |
| Type of therapy (Surgery VS Palliative care) | 2.384** | 2.289-2.483 |
| Type of therapy (Surgery & Chemotherapy VS Palliative care) | 2.046 ** | 1.960-2.135 |
| Type of therapy (Surgery & Radiotherapy VS Palliative care) | 1.722** | 1.452-2.043 |
| Type of therapy (Chemotherapy VS Palliative care) | 1.317 ** | 1.260-1.376 |
| Type of therapy (Radiotherapy VS Palliative care)^c^ | 1.184 ** | 1.098-1.277 |
| Type of therapy (Radiotherapy & Chemotherapy VS Palliative care) | 1.661 ** | 1.541-1.790 |
| Type of therapy (Others VS Palliative care) | 0.828** | 0.765-0.895 |
| Number of episodes per patient | 1.086** | 1.076-1.097 |
| Year (2003 VS 2002) | 1.067** | 1.016-1.122 |
| Year (2004 VS 2002) | 1.145** | 1.089-1.203 |
| Year (2005 VS 2002) | 1.231** | 1.172-1.292 |
| Year (2006 VS 2002) | 1.163** | 1.108-1.221 |
| Year (2007 VS 2002) | 1.211 ** | 1.154-1.270 |
| Year (2008 VS 2002) | 1.223 ** | 1.166-1.281 |
| Year (2009 VS 2002) | 1.334 ** | 1.273-1.399 |
| Year (2010 VS 2002) | 1.442 ** | 1.377-1.511 |
| Year (2011 VS 2002) | 1.490 ** | 1.423-1.561 |
| Number of inpatient days per patient | 1.013 ** | 1.012-1.013 |
| Accompanying diseases (Yes VS NO) | 1.132** | 1.106-1.158 |

Note:

^a^**P<0.01, *P<0.05.

^b^ We include link = log. When we write our model out, log ( μ ) = β_0_ + β_1_x_1_ + ... + β_p_x_p_, where μ is the count we are modeling, and log ( ) defines the link function (i.e., how we transform μ to write it as a linear combination of the predictor variables).

^c^ Goodness of fit for the multivariate GLM model: deviance=0.3652 (p≈1), Pearson Χ^2^=1.253(p≈1), so the goodness of fit for the model is very good.
